# Molecular docking analysis of IL-6 and TNF-alpha with phytochemicals from *Nilavaagai Kiyazham* (decoction) used in Siddha medicine

**DOI:** 10.6026/973206300213575

**Published:** 2025-10-31

**Authors:** Nivetha G, Sri Sakthi Logisha M, Subha V, Sowmiya K, Muthukumar N.J, Mahalakshmi V

**Affiliations:** 1 Department of Varmam Maruthuvam, National Institute of Siddha, the Tamil Nadu Dr. MGR Medical University, Chennai, India; 2 Department of Sirappu Maruthuvam, National Institute of Siddha, the Tamil Nadu Dr. MGR Medical University, Chennai, India; 3 Residential Medical Officer, National Institute of Siddha, Chennai, India; 4 Consultant, Aaruthra Siddha Varmam Clinic, Vadapalani, Chennai, India; 5 Central Council for Research in Siddha, the Tamil Nadu Dr. MGR Medical University, Chennai, India; 6 Department of Siddhar Yoga Maruthuvam, National Institute of Siddha, the Tamil Nadu Dr. MGR Medical University, Chennai, India

**Keywords:** *Nilavaagai Kiyazham*, IL-6 (Interleukin-6) And TNF-Alpha (Tumor Necrosis Factor-Alpha), molecular docking, osteoarthritis, phytocomponents, ADME

## Abstract

Osteoarthritis (OA) is a degenerative musculoskeletal disorder marked by progressive cartilage degradation, synovial inflammation and subchondral bone remodeling, driven by pro-inflammatory mediators such as interleukin-6 (IL-6) and tumor necrosis factor-alpha (TNF-α). While non-steroidal anti-inflammatory drugs (NSAIDs) remain the standard treatment, their long-term use is constrained by adverse effects, necessitating safer, multi-targeted therapeutic alternatives. *Nilavaagai Kiyazham* (decoction), a classical Siddha polyherbal formulation composed of 26 botanicals, has been traditionally indicated for Vadha (gas related) disorders, aligning with osteoarthritic symptomatology. Therefore, it is of interest to assess the binding efficacy of selected phytoconstituents such as *Tinosporide*, *α-tocopherol* and *Apigenin* with IL-6 and TNF-α. The compounds demonstrated high binding affinities with multiple key interactions at active sites, indicating their potential to inhibit inflammatory signaling pathways implicated in OA pathogenesis. Thus, data substantiate the anti-inflammatory claims of *Nilavaagai Kiyazham* and support its integration as a complementary therapeutic modality in OA management, warranting further pharmacodynamics and clinical evaluation.

## Background:

Knee osteoarthritis (KOA) is a common degenerative bone and joint disease, which is caused by the imbalance of degradation synthesis coupling of chondrocytes, extracellular matrix and subchondral bone under the action of mechanical and biological factors. In recent years, due to the increasing rate of aging populations in many countries and changes in life habits, the prevalence of KOA is increasing at an alarming rate. Osteoarthritis affects 7% of the global population, more than 500 million people worldwide, with women disproportionately affected by the condition. 1 The number of people affected globally rose by 48% from 1990 to 2019 and in 2019 osteoarthritis was the 15th highest cause of years lived with disability (YLDs) worldwide and was responsible for 2% of the total global YLDs.1, 2 Although YLDs for osteoarthritis are higher in high Socio-demographic Index (SDI) countries than in middle SDI regions (about 525 vs 220 YLDs per 100000 population), since 1990 the rate of change in YLDs has been far greater in middle SDI countries than in high SDI countries (89% vs 48%) [1, 2]. Osteoarthritis is a leading cause of disability in older adults and the trends of an ageing population and increasing obesity are likely to compound this. These data are concerning but probably underestimate the true size of the problem. Furthermore, the development of treatments for osteoarthritis has not made comparable progress with that for many other musculoskeletal and chronic non-communicable diseases. The standard treatment for osteoarthritis is NSAIDs, which can provide relief from pain and inflammation but have many side effects, so they are only suitable for short-term use. Therefore, there is an urgent need to develop novel strategies for the management of osteoarthritis [3].

Traditional medicine has the potential to provide safe and effective treatments for osteoarthritis. People in search of herbal remedies often look to ancient practises, as many herbal treatments have been in use for centuries. *Nilavaagai Kiyazham* (NVK) is one of the polyherbal formulations from the classical Siddha Varmam literature given for treating painful conditions such as osteoarthritis *Nilavaagai Kiyazham* is comprised of a combination of 26 different herbal ingredients, many of which are known to have anti-inflammatory properties. According to Siddha literature, the symptoms of osteoarthritis are related to the *Azhal keel vaayu*, which is one of the 80 Vadha diseases. Osteoarthritis is considered to be an ailment caused by an imbalance of *Vadha Dosha* in the body [4]. The major ingredient in NVK is Cassia senna, which is traditionally used as a laxative [5]. In the Siddha system of medicine, there is a quote called "*Viresanathal Vadham thaalum*", which means the laxative can reduce the *Vadha Dosha* in the body, which can help to relieve the symptoms of osteoarthritis. According to this quote, using NVK as a laxative can help to restore the balance of *Vadha Dosha* in the body, which in turn can reduce the pain and stiffness associated with osteoarthritis [4, 6]. Although justification made for the use of NVK is based on traditional beliefs, there is still a need for evidence that shows it can have a beneficial effect on osteoarthritis symptoms. Molecular docking analysis can be used to evaluate the bioactivity of NVK and provide an evidence-based approach for assessing its effects on osteoarthritis. Molecular docking has been one of the most basic and important strategies for drug discovery. It allows prediction of molecular interactions that hold together a protein and a ligand in the bound state. This technique has been used to analyse the interactions between NVK and proteins involved in inflammation, thus allowing us to better understand the mechanisms of action behind its potential therapeutic effect on osteoarthritis [7, 8]. Therefore, it is of interest to report the molecular docking-based analysis of selected phytocomponents from NVK against IL-6 and TNF-α, in order to assess their potential role in mitigating the inflammatory pathways associated with osteoarthritis.

## Methodology:

## Ingredients of Nilavaaagai Kiyazham:

The polyherbal formulation NVK consists of 26 raw drugs (Table 1 - see PDF). Each drug has unique pharmacological properties, which when combined in a certain proportion, can provide a better therapeutic effect [8]. The drugs present in NVK are mainly plant-based and most of the ingredients in this formulation possess medicinal properties such as anti-inflammatory, anti-arthritis and antioxidant activities. The effectiveness of NVK can be attributed to the synergistic effect of its herbal components. NVK is a classic example of how multiple medicinal ingredients from plants, with their complementary therapeutic effects, can be combined to create an effective holistic formulation that can provide relief from arthritis pain [4]. NVK contains 26 herbs. It is time consuming to see all the Phytocomponents of the herbs. Hence, the following 6 Phytoconstiuents were selected for docking.

## Protein preparation:

To explore the therapeutic potential of phytocomponents against inflammatory targets IL-6 and TNF-alpha, we retrieved their crystalline structures from the Protein Data BaNVK (PDB) and saved them in PDB format (Figure 1 - see PDF). Using Molegro Molecular Viewer, we cleaned the protein structures by adding essential missing hydrogen atoms and ensuring proper protonation states for accurate docking simulations. Next, we optimized the geometric conformations of the proteins to resolve any steric clashes and create an ideal environment for ligand binding. Docking simulations were performed with AutoDock to evaluate various orientations of the phytocomponents relative to the target proteins. The best docking pose was selected based on a comprehensive interaction analysis, assessing binding affinity and key interactions such as hydrogen bonds and hydrophobic contacts. This thorough preparation is crucial for predicting how phytocomponents may inhibit IL-6 and TNF-alpha, paving the way for potential therapeutic applications in managing arthritis-related inflammation.

## Ligand preparation:

The phytocomponents identified in NVK formulation such as *α-tocopherol*, *Tinosporide*, *Apigenin*, *Vasicinone*, *Ascorbic acid*, *Glabridin* were selected for docking (Table 2 - see PDF and Table 3 - see PDF). Ligand molecules were downloaded from Pubchem [19] in Sdf format. Optimization was done with the force field type MMFF94 using Openable software and saved as pdbqt format and used in docking studies.

## ADME properties prediction:

The ADME (Absorption, Distribution, Metabolism, Excretion and Toxicity) properties of the selected phytocomponents were predicted using the SwissADME online tool. This tool provides a comprehensive analysis of how these compounds may behave in biological systems, aiding in the assessment of their potential efficacy and safety. Additionally, the bioactivity score of the most promising compound was evaluated using Molinspiration, which helps determine its likelihood of exhibiting therapeutic effects.

## Docking:

Docking calculations were performed for the selected phytocomponents alongside standard drugs against the target proteins. Essential hydrogen atoms, Kollman united atom type charges and solvation parameters were incorporated using AutoDock tools. Affinity grid maps were generated with specified grid points and a spacing of 0.375 Å using the AutoGrid program. The docking process utilized AutoDock's parameter set along with distance-dependent dielectric functions to calculate van der Waals and electrostatic interactions. For the docking simulations, the Lamarckian Genetic Algorithm (LGA) and the Solis & Wets local search method were employed. Initial positions, orientations and torsions of the ligand molecules were randomized to enhance exploration, allowing all rotatable bonds to be flexible during the docking process. Each experiment consisted of two independent runs, terminating after a maximum of 250,000 energy evaluations, with a population size of 150. The search utilized a translational step of 0.2 Å, along with quaternion and torsion steps of 5 Å, ensuring thorough sampling of potential binding conformations.

## Results:

In the assessment of the six phytocomponents, *Tinosporide* and *α-tocopherol* exhibited the highest binding potential, each displaying four significant interactions with the IL-6 receptor (Table 4 - see PDF, Table 6 - see PDF). This level of interaction is comparable to that of the standard drug, suggesting that both *Tinosporide* and *α-tocopherol* may effectively inhibit IL-6 activity, which is critical in the inflammatory pathways associated with arthritis. Their structural characteristics likely enable these robust interactions, indicating a potential for these compounds to serve as effective therapeutic agents in managing inflammation. IL-6 contributes to the chronic inflammatory response seen in arthritis, driving processes such as cartilage degradation, joint damage and pain [53, 54]. Following closely, *Apigenin* also demonstrated significant interactions with the IL-6 receptor, though with slightly fewer interactions than *Tinosporide* and *α-tocopherol*. This compound's ability to engage with key binding sites further underscores its potential role in modulating IL-6-mediated inflammatory responses. Given *Apigenin*'s established biological activity, its interactions could contribute to a reduction in inflammatory symptoms and support overall joint health in arthritis patients [55]. Overall, the significant binding of *Tinosporide*, *α-tocopherol* and *Apigenin* to the IL-6 receptor highlights their potential as natural anti-inflammatory agents. These findings warrant further investigation into their therapeutic mechanisms and efficacy in clinical settings, particularly as complementary treatments alongside conventional therapies for arthritis. Similarly, in the evaluation of interactions with the TNF-alpha receptor, *Apigenin* emerged as a standout phytocomponent, showcasing four significant interactions. This level of binding affinity positions *Apigenin* as a promising candidate for effectively inhibiting TNF-alpha, which plays a pivotal role in mediating inflammatory responses in arthritis (Table 5 - see PDF and Table 7 - see PDF).

The structural attributes of *Apigenin* may facilitate these interactions, thereby enhancing its therapeutic potential [56]. Following *Apigenin*, other compounds, including *α- tocopherol*, *Tinosporide*, *Vasicinone*, *Ascorbic acid* and *Glabridin*, also demonstrated meaningful interactions with the TNF-alpha receptor, although with slightly fewer binding events. Each of these compounds contributes to the inhibition of TNF-alpha activity, suggesting that they may collectively play a role in mitigating the inflammatory processes associated with arthritis. The presence of multiple hydrogen bond donors and acceptors in these compounds likely enhances their binding efficacy, indicating their potential for therapeutic application [57, 55]. The significant interactions of these phytocomponents with the TNF-alpha receptor highlight their potential to serve as effective anti-arthritis agents. Their ability to modulate TNF-alpha activity not only emphasizes the importance of these natural compounds in inflammatory disease management but also points toward a potential pathway for the development of new therapeutic strategies. Further research into their pharmacodynamics, optimal dosing regimens and possible synergistic effects when used in conjunction with traditional anti-inflammatory drugs is warranted. In conclusion, the binding interactions observed in both IL-6 and TNF-alpha receptors underscore the therapeutic promise of *Tinosporide*, *α-tocopherol* and *Apigenin*, among others. Their potential to exert anti-arthritis effects positions them as valuable candidates for further exploration in the quest for effective, natural treatments for inflammatory conditions.

## Conclusion:

The therapeutic potential of NVK in osteoarthritis management, supported by molecular docking analyses of its key phytocomponents is shown. Compounds such as *Tinosporide*, *α-tocopherol* and *Apigenin* demonstrated strong binding affinities with pro-inflammatory targets IL-6 and TNF-α, suggesting their role in modulating inflammatory pathways. Thus, we show offer scientific validation for the traditional use of *Nilavaagai Kiyazham* and support its potential as a complementary anti-inflammatory therapy. Further studies are warranted to explore its clinical efficacy, mechanisms of action and integration into holistic arthritis treatment strategies.

## Ethical approval:

The authors declare that due to a literature review this study does not need any ethical approval.

## Consent to participate:

Not applicable.

## Consent to publish:

Not applicable.

## Author contributions:

All authors have equally contributed in the manuscript preparation like concept, designing and collection of the data, as well as the writing and revision of the article.

## Funding:

The authors declare that no funds, grants, or other support were received during the preparation of this manuscript.

## Availability of data and Materials:

All the data's are available with authors upon reasonable request.

## Source of funding:

No funds, grants, or other support was received for this work.
